# Some lessons from the COVID-19 pandemic virus

**DOI:** 10.1590/1516-3180.2020.138320052020

**Published:** 2020-06-22

**Authors:** Isabela Martins Benseñor, Paulo Andrade Lotufo

**Affiliations:** I MD, PhD. Full Professor, Department of Internal Medicine, Faculdade de Medicina FMUSP, Universidade de Sao Paulo, São Paulo (SP), Brazil.; II MD, DrPH. Full Professor, Department of Internal Medicine, Faculdade de Medicina FMUSP, Universidade de Sao Paulo, São Paulo (SP), Brazil.

When the spread of the infection caused by the new coronavirus named COVID-19 was officially declared by the World Health Organization (WHO) to be a pandemic, the first idea was that it was just another respiratory disease related to a new virus strain; just another flu-like syndrome. The clinical presentation would have a classical spectrum ranging from asymptomatic infection to a respiratory distress syndrome needing respiratory support in intensive care units in the most severe cases. Therefore, the idea was that the impact of the virus would be restricted to the upper and lower respiratory system.

The development of the pandemic has shown us the real face of the virus. It is more complex and more particular than was expected by healthcare professionals and stakeholders. That was the first lesson that the virus has given to us.

The symptoms of the infection have multiple facets and it is not simple to fight against it. First, the key receptors enabling entry for the virus are angiotensin-converting enzyme 2 (ACE2) receptors, which have a strong relationship with blood pressure homeostasis through the renin-angiotensin-aldosterone system.^1^ At the same time, the descriptions of the Wuhan series of cases have been showing that people with a previous diagnosis of hypertension presented an unexpectedly high risk of development of severe cases of COVID-19. Moreover, the virus has been found to have direct action at the endothelial level, thus indicating that the infection could be acting on unstable atherosclerotic plaques in the coronary artery bed, and hence increasing the risk of sudden cardiac death, myocardial infarction and decompensated heart failure.^2^^,^^3^

Moreover, the infection has been found to be associated with thrombosis and thromboembolic episodes, not only in the lungs but also in relation to stroke and other venous and arterial thromboembolic complications with a high risk of death.^4^^,^^5^ Regarding stroke, large-vessel stroke has been described as a presenting feature of COVID-19 in young adults.^6^ Other complications that have been reported include septic and cardiovascular shock,^7^ especially in people with a history of comorbidities;^8^ and also alterations in kidney function. Unusual presentations, such as mesenteric thrombosis and acute thyroiditis, among others, need further investigation.

The second lesson from COVID-19 is that the relationship between humankind and the surrounding environment needs to undergo changes, urgently. Most of the more recent pandemics have probably arisen as the consequence of an unhealthy relationship between humans and wild animals and livestock.

We are constantly invading the space of other living animals. This is not new. We have forgotten, in the history of other diseases, that many of them came from animals that humans have domesticated, starting in the Neolithic age, more than 10,000 years ago. The agricultural era emerged accompanied by domestication of wild animals. Influenza originated from pigs and chicken; common cold was brought by horses; tuberculosis from cattle; and Lyme disease from infected ticks that live mostly in deer.^9^ More recently, AIDS originated from chimpanzees; SARS coronavirus probably from civets and Asian raccoon dogs (*Nyctereutes procyonoides*),^10^ swine influenza (H1N1) from pigs^11^ and Spanish influenza probably also from pigs.^12^

Another lesson that we need to understand is that no city and no country is prepared to care for so many patients that need ventilatory support at the same time. One of richest cities in the world, New York, has had to accept the limitations of its healthcare system in this situation. However, it is important to state that the burden of the disease will be greater in low to middle-income countries with their overloaded healthcare systems and high numbers of people living in poor communities. In addition, one fact is clear: Asian countries have been more successful in dealing with COVID-19 than most Western countries.^13^

The COVID-19 pandemic will not end with the discharge of the last patients in whichever place in the world where this occurs. Patients who have stayed in an intensive care unit for many weeks will need rehabilitation, physiotherapy and speech therapy, and probably mental health support after discharge. The real impact of the pandemic on mental health is not known at this point in time.

The pandemic has changed the way in which people die. Patients have mostly died alone, far from their families. This is and will be a great burden for these families that lost relatives without the proper rites of passage, such as the viewing and funeral ceremonies. Most families are not given the chance to give a proper farewell to beloved relatives and friends. It is important to think about this. Moreover, COVID-19 is just the most recent pandemic: it is unlikely to be the last one.

We need to become prepared for new pandemics and to create new scientific protocols and rites of passage to deal with all the biological and spiritual consequences of this disease that has devastated so many countries in 2020, a year that we will not forget. We need to learn from this and seek new solutions to deal with future pandemics.
